# An approach to assess and adjust for the influence of multicollinear covariates on metabolomics association patterns—applied to a study of the associations between a comprehensive lipoprotein profile and the homeostatic model assessment of insulin resistance

**DOI:** 10.1007/s11306-022-01931-6

**Published:** 2022-09-02

**Authors:** Olav M. Kvalheim, Tarja Rajalahti, Eivind Aadland

**Affiliations:** 1 Department of Chemistry, University of Bergen, Bergen, Norway; 2 Førde Health Trust, Førde, Norway; 3 Red Cross Haugland Rehabilitation Centre, Flekke, Norway; 4 Department of Sport, Food and Natural Sciences, Western Norway University of Applied Sciences, Sogndal, Norway

**Keywords:** Insulin resistance, HOMA, Lipoprotein subclasses, Adiposity, Physical activity, Covariate projections

## Abstract

**Introduction:**

Comprehensive lipoprotein profiling using proton nuclear magnetic resonance (NMR) spectroscopy of serum represents an alternative to the homeostatic model assessment of insulin resistance (HOMA-IR). Both adiposity and physical (in)activity associate to insulin resistance, but quantification of the influence of these two lifestyle related factors on the association pattern of HOMA-IR to lipoproteins suffers from lack of appropriate methods to handle multicollinear covariates.

**Objectives:**

We aimed at (i) developing an approach for assessment and adjustment of the influence of multicollinear and even linear dependent covariates on regression models, and (ii) to use this approach to examine the influence of adiposity and physical activity on the association pattern between HOMA-IR and the lipoprotein profile.

**Methods:**

For 841 children, lipoprotein profiles were obtained from serum proton NMR and physical activity (PA) intensity profiles from accelerometry. Adiposity was measured as body mass index, the ratio of waist circumference to height, and skinfold thickness. Target projections were used to assess and isolate the influence of adiposity and PA on the association pattern of HOMA-IR to the lipoproteins.

**Results:**

Adiposity explained just over 50% of the association pattern of HOMA-IR to the lipoproteins with strongest influence on high-density lipoprotein features. The influence of PA was mainly attributed to a strong inverse association between adiposity and moderate and high-intensity physical activity.

**Conclusion:**

The presented covariate projection approach to obtain net association patterns, made it possible to quantify and interpret the influence of adiposity and physical (in)activity on the association pattern of HOMA-IR to the lipoprotein features.

**Supplementary Information:**

The online version contains supplementary material available at 10.1007/s11306-022-01931-6.

## Introduction

The homeostatic model assessment of insulin resistance (HOMA-IR) (Matthews et al., 1985; Muniyappa et al., 2007) is derived from fasting insulin and glucose. This measure can be obtained from frozen blood samples and is therefore commonly used for assessing insulin resistance (IR) in epidemiological studies. Many lipoprotein subclasses correlate to IR (Garvey et al., 2003; Goff et al., 2005). Comprehensive lipoprotein profiles can be derived from high-throughput proton nuclear magnetic resonance (NMR) spectroscopy of blood samples. Thus, Shalaurova et al. (2014) derived a lipoprotein IR index from the associations of HOMA-IR to the concentrations of large very-low density lipoproteins (VLDL), small low-density lipoproteins (LDL) and large high-density lipoprotein (HDL) particles, and the average size of VLDL, LDL, and HDL particles. Their lipoprotein IR index was based on a cohort of almost 5000 nondiabetic subjects and independently validated in a cohort consisting of insulin sensitive, insulin resistant and untreated diabetic subjects using the glucose disposal rates (GDRs) (Muniyappa et al., 2007).

Adiposity associates with both lipoproteins and IR and is therefore a covariate influencing this association. For example, Okuma et al. (2013) observed an inverse association of visceral obesity and HOMA-IR with an HDL subclass pattern of very large, large, and intermediate HDL particles in Japanese schoolchildren. Physical activity (PA) also associates both with lipoproteins and IR (Krekoukiaa et al., 2007; Phillips et al., 2018). Association patterns between PA descriptors and lipoprotein profiles from exercise interventions studies (Kraus et al., 2002; Sarzynski et al., 2015) and observational studies of associations between measures of leisure-time PA and lipoproteins (Bell et al., 2018; Kujala et al., 2013) display the same overall picture: A positive association of PA to concentration of HDL, large HDL and large LDL particles, average size of HDL and LDL particles, and, an inverse association of PA to concentration of lipoprotein triglycerides (TG), VLDL, large VLDL and small LDL particles, and the average size of VLDL particles. Thus, lifestyle factors, as reflected in adiposity and PA, influence on the associations between lipoproteins and IR.

While the influence of adiposity and PA on the association pattern between HOMA-IR and the lipoprotein profile has been qualitative inferred, quantitative assessment is limited. This is partly due to the difficulties posed by the inverse relationship of PA and adiposity to the lipoprotein profile (Rajalahti et al., 2021a), which imply that physical inactivity correlates to adiposity, and, accordingly, that it is difficult to separate their influence on the association pattern of IR with the lipoproteins. But quantification has also been hampered by the lack of methods to handle linearly dependent high-resolution PA descriptors derived from accelerometric measurements (Aadland et al., 2019). Recently, we developed a strategy to assess the independent and joint influence of multicollinear descriptors of adiposity and PA on the association pattern of aerobic fitness to lipoproteins. We decomposed the multivariate PA and adiposity descriptors by principal component analysis (PCA) (Bro & Smilde, 2014) and projected both the outcome and the explanatory variables on the principal component score vectors to obtain net association patterns (Rajalahti, 2021a, 2021b). A drawback of this approach is that interpretation is complicated by the need for many principal components to present a covariate descriptor. An alternative approach would be to regress the outcome on the multicollinear PA and adiposity descriptors using partial least squares (PLS) (Wold et al., 1984) followed by target projection (Kvalheim & Karstang, 1989; Rajalahti & Kvalheim, 2011) to obtain single predictive score vectors for PA and adiposity and project on the target score vectors. This approach facilitates the interpretation of the influence of covariates. In this paper, we aim at developing this approach to obtain “net” association patterns to assess quantitively the influence of adiposity and PA on the association pattern between HOMA-IR and the lipoprotein profile.

## Assessment and adjustment for covariates to obtain net associations

We provide a general approach to investigate how the association pattern between an outcome variable *y* and a set of explanatory variables, {*x*_1_, *x*_2_, …, *x*_M_} is influenced by and can be adjusted for covariates, {*z*_1_, *z*_2_, …, *z*_M_}. Such covariates can be, but are not limited to, confounders affecting the association of the outcome to the explanatory variables.

The term net association pattern is used to imply the pattern obtained after removal of some or all covariates. The vector **y** contains the measurements for *y* and the matrices **X** and **Z**, the corresponding measurements for the *x*-variables and *z*-variables, respectively.

For ordinary multiple linear regression (MLR) analysis, explanatory variables (including covariates) are mutually adjusted by their inclusion in a joint statistical model, given that this model allows for interpretation of the explanatory variables´ independent associations with the outcome. However, this procedure is *not* suited for multicollinear descriptors, where associations are not independent, but collinear. To handle this situation, adjustment for covariates can be accomplished by calculating a regression model between the outcome and the covariates:

1a $${\mathbf{y}} = {\mathbf{Zb}}_{{{\text{Z}},{\text{y}}}} + {\mathbf{e}}_{{\text{y}}}$$

The outcome is adjusted for the covariates by using the residuals **e**_y_ in further analysis (Aadland et al., 2019).

Alternatively, the explanatory variables can be adjusted for the covariates. For each explanatory variable **x**_i_, one calculates

1b $${\mathbf{x}}_{{\text{i}}} = {\mathbf{Zb}}_{{{\text{Z}},{\text{xi}}}} + {\mathbf{e}}_{{{\text{x}},{\text{i}}}} \left\{ {{\text{i}} = {1},{ 2}, \, \ldots ,{\text{M}}} \right\}$$

and the residuals **e**_x,i_ are used as explanatory variables in subsequent analyses.

Traditionally, MLR is used to calculate the regression vectors from Eqs. (1a) and (1b) needed to adjust either the outcome, the explanatory variables or both for covariates. If the covariates possess linear dependency, the calculation of a Moore–Penrose inverse (Rao & Mitra, 1971) represents a solution to relate the covariates to the outcome or the explanatory variables:

2a $${\mathbf{b}}_{{{\text{Z}},{\text{y}}}} = {\mathbf{Z}}^{ - } {\mathbf{y}}$$

2b $${\mathbf{B}}_{{{\text{Z}},{\text{X}}}} = {\mathbf{Z}}^{ - } {\mathbf{X}}$$

Superscript—implies the Moore–Penrose inverse, i.e. **Z**^−^** = **(**Z**^T^**Z**)^−1^**Z**^T^. As shown by Rajalahti et al., (2021a, 2021b), PCA can be used for handling the situation posed by linear dependent covariates but other projection methods such as partial least (PLS) are available. We will now explore some of the possibilities.

By using a general projection algorithm (Kvalheim, 1987; Rajalahti & Kvalheim, 2011), it is possible to eliminate the influence of covariates on both the outcome **y** and the explanatory variables **X** simultaneously. For didactic reasons, we first consider the trivial case for a single covariate z. Collect the column-centred vectors **z** and **y** for the covariate and the outcome, respectively, and the column-centred matrix **X** in the augmented matrix **X**_aug_ = [**z y X**].

A covariate projection (CP) to assess and adjust for a single covariate consists of four steps:

i. define the CP through a weight vector **w**_CP_ with all elements equal to zero except the element corresponding to the position of the covariate in **X**_aug_, i.e., the first element of **w**_CP_ which is given the value one.
ii. calculate the CP score vector **t**_CP_ = **X**_aug_**w**_CP_. For the case of a single covariate **t**_CP_ = **z**.
iii. calculate the CP loading vector **p**_CP_^T^ = **t**_CP_^T^**X**_aug_/(**t**_CP_^T^**t**_CP_)
iv. obtain the adjusted augmented residual matrix as **E**_**aug**_ = **X**_**aug**_ – **t**_CP_**p**_CP_^T^

The column in the residual matrix **E**_aug_ corresponding to the outcome variable is **e**_y_ = **y** − **y**(**z**^T^**y)**/(**z**^T^**z**) which is exactly the residuals of **y** obtained by regressing the outcome on the covariate. Similarly, the residual vectors of the *x*-variables after CP on the covariate are **e**_x,i_ = **x**_i_ − **x**_i_(**z**^T^**x**_i_)/(**z**^T^**z**) for *i* = 1,2, …,M. The column in **E**_**aug**_ representing the residuals of the covariate after CP, is a column vector where all elements are zero, **e**_z_ = **z** − **z**(**z**^T^**z**)/(**z**^T^**z**) = **0**. Thus, for a single covariate, the residual matrix **E**_aug_ contains the adjusted (“net”) outcome and explanatory variables and a column of zeros for the covariate.

Generalization to several covariates not being linearly dependent, is straightforward: Add one column for each covariate to obtain the matrix [**Z y X**]. After CP for the first covariate, repeat the CP procedure on the resulting residual matrices **E**_aug_. This procedure continues for the updated residual matrix resulting from repeating steps i–iv in the algorithm above until every covariate has been used in the CP algorithm. After this procedure, the elements in **E**_aug_ are zero for all the covariates and contain adjusted outcome and explanatory variables from which we can calculate net association patterns between outcome and explanatory variables by regression.

Removal of the subspace spanned by the covariates from either the outcome variable or the explanatory variables lead to the same regression model in the subsequent regression of the outcome on the explanatory variables. However, as discussed below, it is better to adjust both outcome and explanatory variables jointly using the projection algorithm. This allows interpretation and visualization of the influence of covariates within a “global” joint model composed of two orthogonal parts: One part describing the variance pattern of the outcome and the explanatory variables shared with the covariates, and another part describing the net association pattern between the adjusted outcome and explanatory variables.

In case of linear dependent covariates, we cannot use the CP algorithm directly but proceed via the calculation of a latent variable model representing the covariates. Recently, we decomposed strongly multicollinear and even linear dependent covariates using PCA and used the orthogonal principal score vectors in the CP algorithm to isolate the influence of these covariates (Rajalahti et al., 2021a, 2021b). Another possibility is to model the relation between the outcome and the covariates by PLS and then use the PLS score vectors in the CP algorithm. The drawback for both PCA and PLS is that many latent variables are usually needed to describe a multivariate covariate. The physical activity descriptor in our application represents an example of this situation. This makes interpretation and visualization difficult. To circumvent the problem, we therefore propose to post-process the validated PLS model between an outcome and a multivariate covariate using target projection (Kvalheim & Karstang, 1989; Rajalahti & Kvalheim, 2011). By this procedure a single predictive target component is obtained that contains the predictive information in the PLS model. The general projection algorithm provides the target component for the multivariate covariate *a* by using the normalized regression vector **b**_Za,y_ as weight vector, i.e., **w**_TP,a_ = **b**_Za,y_/‖**b**_Za,y_‖. The target scores maximally correlate to the predicted outcome. Thus, **t**_TP_ = **Z**_a_**w**_TP_ = **Z**_a_**b**_Za,y_/‖**b**_Za,y_‖. By projecting **Z**_a_ on the target score vector, the target loadings **p**_TP,a_ = **Z**_a_^T^**t**_TP,a_ /(**t**_TP,a_^T^t_TP,a_) are obtained and the target model for the multivariate covariate can be formulated:

3a $${\mathbf{Z}}_{{\text{a}}} = {\mathbf{t}}_{{{\text{TP}},{\text{a}}}} {\mathbf{p}}_{{{\text{TP}},{\text{a}}}}^{{\text{T}}} + {\mathbf{E}}_{{{\text{TP}},{\text{a}}}}$$

3b $${\mathbf{y}} = {\mathbf{t}}_{{{\text{TP}},{\text{a}}}} \left\| {{\mathbf{b}}_{{{\text{Za}},{\text{y}}}} } \right\| + {\mathbf{e}}_{{{\text{Za}},{\text{y}}}}$$

The standardized score vector **t**_TP,a_ is subsequently used in the CP algorithm to adjust outcome and explanatory variables for the multivariate covariate *a*. This simplifies interpretation compared to using many principal or PLS components for describing linear dependent covariates.

Note that although all covariates can be incorporated in a single PLS model and thus be assessed and adjusted for jointly by a single target projection model, we partition the covariates into groups and use the CP algorithm stepwise to be able to separate the influence of the different groups of covariates on the association pattern. Thus, the multivariate physical activity and adiposity covariates as well as the univariate covariates age and sex are treated separately in the CP algorithm in the application in this work. This approach enables interpretation of the association patterns of the outcome to the covariates together with the net (residual) variance in the explanatory variables and the outcome in a variance plot (Rajalahti et al., 2021a).

We include covariates, outcome, explanatory variables and target score vectors representing multivariate covariates in the variance plot for visualization and interpretation of the partition of variance for all variables. The decomposition can be written as:

4 $$[{\mathbf{T}}_{{\text{Z}}} {\mathbf{ZyX}}] = \, \sum {\mathbf{t}}_{{{\text{CP}},{\text{a}}}} {\mathbf{p}}_{{{\text{CP}},{\text{a}}}}^{{\text{T}}} + {\mathbf{E}}_{{[{\text{Tz}},{\text{Z}},{\text{y}},{\text{X}}]}}$$

The matrix **T**_Z_ contains standardized target score vectors for the multivariate covariates, while **E**_[Tz,Z,y,X]_ contains the net values of [**T**_Z_**ZyX**] after adjusting for the covariates. The net values for the outcome, **y**_net_, and the explanatory variables, **X**_net_, in this matrix can subsequently be used to obtain the net associations pattern between outcome and explanatory variables by PLS regression followed by post-processing to obtain a single target component displaying the predictive association pattern:

5a $${\mathbf{X}}_{{{\text{net}}}} = {\mathbf{t}}_{{{\text{TP}},{\text{net}}}} {\mathbf{p}}_{{{\text{TP}},{\text{net}}}}^{{\text{T}}} + {\mathbf{E}}_{{{\text{TP}},{\text{net}}}}$$

5b $${\mathbf{y}}_{{{\text{net}}}} = {\mathbf{t}}_{{{\text{TP}},{\text{net}}}} \left\| {{\mathbf{b}}_{{{\text{net}}}} } \right\| + {\mathbf{e}}_{{{\text{y}},{\text{net}}}}$$

From Eqs. (5a) and (5b), selectivity ratios (SRs) (Rajalahti et al., 2009) can be calculated quantifying the predictive information in the *x*-variables. SR for a variable is defined as the ratio of explained variance (by the TP model) to residual variance:

6 $${\text{SR}}_{{\text{i}}} = \, \left\| {{\mathbf{t}}_{{{\text{TP}},{\text{net}}}} {\text{p}}_{{{\text{i}},{\text{ TP}},{\text{net}}}} } \right\|^{{2}} /\left\| {{\mathbf{e}}_{{{\text{i}},{\text{TP}},{\text{net}}}} } \right\|^{{2}} \left\{ {{\text{i}} = {1},{ 2}, \, \ldots ,{\text{M}}} \right\}$$

The SRs can be used for interpretation and visualization in an SR plot. Such plots display the overall predictive association patterns between outcome and explanatory variables and rank the explanatory variables according to predictive importance. As shown in the result section, SR plots can be built from models during various stages of adjustment to provide quantitative information about the influence of covariates on the association pattern.

## Materials and methods

### Population

We used baseline data for children participating in the Active Smarter Kids study in this work (Resaland et al., 2015). 1129 5th graders (94% of those invited) from 57 schools in Western Norway participated in the study. Of these, 841 were included in the present work. The inclusion criterion was that the children had complete and valid data for all the variables described below, i.e., the lipoprotein profile, insulin, glucose, the physical activity intensity spectrum, and three measures of adiposity.

### Blood samples

Overnight fastening serum samples were obtained and stored at − 80 °C according to a standard protocol (Lin et al., 2016) and shipped on dry ice to the laboratories for the blood analyses.

### HOMA-IR

The Endocrine Laboratory of the VU University Medical Center (VUmc; Amsterdam, the Netherlands) measured insulin and glucose. HOMA-IR was calculated as fastening serum insulin times fasting serum glucose divided by 22.5 (Matthews et al., 1985). The product of fasting plasma insulin of 5 μU/ml and normal fasting plasma glucose of 4.5 mmol/l is 22.5. This value represents an individual with “normal” insulin sensitivity and a HOMA-IR score equal to 1 (Muniyappa et al., 2007).

### Lipoproteins

The serum lipoprotein profile was predicted from proton NMR spectra as described by Rajalahti et al. (2021a, 2021b). The profile is characterized by 26 measures: Concentrations of total cholesterol (TC), total triglyceride (TG), chylomicrons (CM), very low density lipoproteins (VLDL), low density lipoproteins (LDL), high density lipoproteins (HDL), two subclasses of CM (CM-1 and CM-2), five subclasses of VLDL (VLDL-L1, VLDL-L2, VLDL-L3, VLDL-M, VLDL-S), four subclasses of LDL (LDL-L, LDL-M, LDL-S, LDL-VS), six subclasses of HDL (HDL-VL1, HDL-VL2, HDL-L, HDL-M, HDL-S and HDL-VS), and the average particle size of VLDL, LDL and HDL subclasses. The abbreviations VL, L, M, S and VS imply very large, large, medium, small, and very small particles. TG and cholesterol lipoprotein features were separately and independently calculated from in-house developed and validated PLS models with reference values from liquid chromatography (Okazaki et al., 2005) for all subclasses, and then combined to obtain features representing the total concentration for each subclass of lipoproteins. Fractions of TG and cholesterol subclasses were used to calculate average particle size for VLDL, LDL and HDL.

### Adiposity measures

We used three measures of adiposity: Body mass index (BMI) calculated as mass divided by the squared height (kg/m^2^), waist circumference to height (WC/H), and skinfold thicknesses (sum of biceps, triceps, subscapular, and suprailiac thicknesses). Details of measurements can be found in Rajalahti et al., (2021a, 2021b).

### Physical activity data

PA was measured using the ActiGraph GT3X + accelerometer (Pensacola, FL, USA) (John & Freedson, 2012) worn at the right hip over seven consecutive days, except during water activities (swimming, showering) or while sleeping. We derived a PA descriptor of time (minute/day) spent in 23 intensity intervals from the measurements on the vertical axis to obtain a PA intensity spectrum (Aadland et al., 2019). The intensity intervals used for the PA descriptor were 0–99, 100–249, 250–499, 500–999, 1000–1499, 1500–1999, 2000–2499, 2500–2999, 3000–3499, 3500–3999, 4000–4499, 4500–4999, 5000–5499, 5500–5999, 6000–6499, 6500–6999, 7000–7499, 7500–7999, 8000–8499, 8500–8999, 9000–9499, 9500–9999 and ≥ 10,000 counts per minute (cpm).

### Pretreatment of data

The repeated Monte-Carlo resampling method used to validate the number of PLS components with predictive information produces more stable models if the variables are approximately normally distributed (Kvalheim et al., 2018). All variables, except age and sex, were thus log-transformed. After log transformation, normal probability plots showed that only TG, CM, VLDL and a few CM and VLDL subclasses deviated from normal distribution. The data are listed as Suppl. Mat. 1.

Prior to further statistical analysis, the data were mean-centered and standardized to unit variance. Also TP score vectors were standardized.

### Procedure for deriving “net” data

It is well-known that age and sex influence the lipoproteins (Rajalahti et al., 2016). Therefore, all the variables were adjusted for these two confounders using the CP algorithm. With the purpose of revealing the influence of adiposity and PA or both on the associations of lipoproteins (explanatory variables) to HOMA-IR (outcome), additional projections were performed for these covariates. The three adiposity measures were strongly multicollinear, while the PA variables were linear dependent. Therefore, projections for PA and adiposity to obtain net HOMA-IR and lipoprotein variables were performed by using the target component score vectors in the covariate projection procedure. Separate PLS models between HOMA-IR and the adiposity and PA descriptors were built for an increasing number of components using repeated Monte Carlo resampling with 1000 repetitions leaving out 50% of the data for predictions. The number of predictive PLS components was selected by locating the minimum of the root-median-squared-error-of-prediction for the PLS models and additionally requiring that the median prediction error for the model should be significantly lower than for the model with one PLS component less (Kvalheim et al., 2018). Target projections for the validated PLS models showed that the target scores explained 42.6% and 79.2% of the total variance in PA and adiposity variables, respectively, and 11.4 and 27.7% in HOMA-IR for PA and adiposity, respectively. The standardized target scores were subsequently used in the projection algorithm to assess and adjust for the influence of adiposity and PA individually and jointly on the net associations. As shown above, target projection embraces all the *predictive* information in the validated PLS regression models.

### Modelling and visualization of “net” association patterns

We calculated the net association patterns of HOMA-IR to the lipoprotein features in three steps:

1. PLS regression with 1000 repetitions of Monte Carlo resampling to establish predictive models of HOMA-IR to the lipoproteins using the same model selection procedure as above for relating HOMA-IR to the covariates adiposity and PA.
2. Target projection to quantify and detach the influence of adiposity or PA or both on the association pattern of HOMA-IR to the lipoproteins.
3. Transformation of these patterns into selectivity ratios (Rajalahti et al., 2009) to interpret the influence of PA and adiposity on the association pattern of HOMA-IR to the lipoproteins.

## Results and discussion

Table 1 summarizes features of the regression models calculated for the association between HOMA-IR and lipoproteins.

**Table 1:** Remaining variance and explained variances of HOMA-IR and lipoproteins after adjustments

Variables adjusted for	Var_adj_ (HOMA-IR)^a^	Var_adj_ (LP)^a^	R2LP^b^	R2HOMA^b^	SR plot
Age and sex	96.7	98.6	35.9	20.2	Figure 1a
Age, sex, and adiposity	72.2	91.8	28.0	9.8	Figure 1b
Age, sex, and PA	87.4	95.5	32.8	14.4	Figure 1c
Age, sex, adiposity, and PA	70.7	91.0	27.2	8.9	Figure 1d

^a^Percent remaining variance of total variance in HOMA-IR and lipoproteins (LP) after adjustments

^b^Percent explained variance in lipoproteins (LP) and HOMA-IR of their original total variance before adjustments

Data adjusted for (a) age and sex, (b) age, sex, and adiposity, (c) age, sex, and PA, and (d) age, sex, adiposity, and PA.

**Fig. 1:**
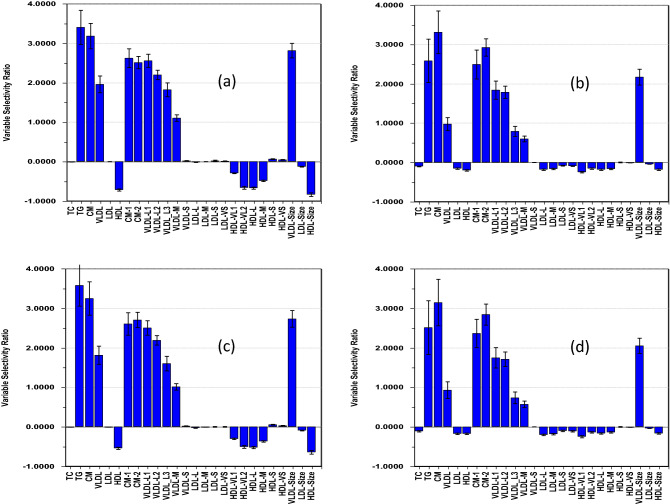
Selectivity ratio plots of regression models using HOMA-IR as outcome and the 26 lipoprotein features as explanatory variables

Since the age variation is narrow and prepubertal boys and girls have similar lipoprotein profiles (Rajalahti et al., 2016), the confounders age and sex have only small effects on the variances in HOMA-IR and lipoproteins (Table 1, first row) and thus on the net association pattern between HOMA-IR and lipoproteins. The corresponding SR plot of the model obtained after adjustment for these confounders (Fig. 1a) displays a strong positive association pattern between HOMA-IR and the triglyceride rich lipoprotein classes of CM and VLDL and the average VLDL particle size. Moderate negative associations are observed between HOMA-IR and HDL, the HDL subclasses of very large, large, and medium size particles, and the average size of HDL particles. No associations are observed with the subclass VLDL-S and the LDL features. Overall, this pattern resembles previous findings for adiposity in children (Resaland et al., 2018) and is almost inversely associated with the pattern we found for PA (Rajalahti et al., 2021a) and aerobic fitness (Rajalahti et al., 2021b). The inverse associations of HOMA-IR to large and very large HDL particles coincide with the findings of Okuma et al. (2013) in Japanese schoolchildren for the association of visceral obesity to HDL subclasses. Furthermore, the inverse association between HOMA-IR and HDL echoes the finding of Blackett et al. (2005) of obesity-related lowering of HDL cholesterol already present in 5–9 years old Cherokee Indian children. Our results also mostly comply with the association pattern to HOMA-IR and BMI in 61 obese adolescents observed by Slyper et al. (2014). The patterns agree for TG, VLDL and large VLDL particles and HDL and their subclasses but deviate for LDL and VLDL-S particles, the latter which are often termed intermediate-density lipoproteins (IDL). The discrepancies may be due to differences in age group between the two studies since the lipoprotein pattern changes during puberty (Dai et al., 2009; Freedman et al., 2004; Labarthe et al., 2003; Stozicky et al., 1991). In summary, the association pattern found in our cohort of children mainly agrees with previous investigations for children, but deviates for LDL features observed for adolescents, but this may be attributed to the impact of puberty on the lipoprotein profile.

Adjustment by adiposity target scores in the projection algorithm reduces the original variance in HOMA-IR and lipoprotein features by 24.5% and 6.8%, respectively, with an accompanying halving of the variance explained in HOMA-IR (Table 1, row 2). The corresponding SR plot (Fig. 1b) shows that this is due to a strong weakening of the inverse associations of HOMA-IR to the HDL features and weakening of the positive associations to the VLDL features and total TG.

Adjustment instead by PA target scores reduces the variance in HOMA-IR and lipoproteins by 9.3% and 3.1%, respectively (Table 1, row 3). This is less than half of what was observed for adiposity. The reduction in explained variance of HOMA-IR is also approx. half of that observed for adjustment by adiposity leading to much smaller changes in the association pattern (Fig. 1c).

Furthermore, very little additional variance is removed from HOMA-IR and the lipoproteins by adjusting for both adiposity and PA (Fig. 1d) compared to adjustment for only adiposity (Fig. 1b) and the association pattern is only marginally affected. Thus, the influence of adiposity on the association pattern is much stronger than that for PA. The much weaker influence of PA on the association pattern after first adjusting for adiposity compared to the result observed without adjusting for adiposity suggests a strong relation between adiposity and PA. This was verified by calculating a PLS model between the adiposity target component and the PA descriptor consisting of 23 intensity ranges. The model explained 23.7% of the variance in adiposity and the SR plot (not shown) revealed an increasingly stronger inverse association of adiposity to PA intensity peaking around 7500–8000 cpm. Thus, PA is indirectly partially adjusted for when we adjust for adiposity as also indicated by previous findings in this cohort (Rajalahti et al., 2021a).

Other methods for variable importance are available to study association patterns and comparative studies have been performed (Farrés et al., 2015; Mehmood et al., 2020). Variable importance in projection (VIP) is a commonly used method to study metabolomics association patterns. For comparison, we have included VIP plots corresponding to the SR plots (Supplementary Material 2). The VIP plots show the same strong weakening of the associations of HOMA-IR to the HDL features as the SR plots accompanying adjustment for adiposity target scores. However, the weakening in the associations of HOMA-IR to TG and the triglyceride-rich lipoproteins visualized in the SR plots (Fig. 1b, d) is not observed in the corresponding VIP plots. Thus, for TG and the triglyceride-rich lipoproteins, the VIP plots do not comply to previous investigations (Slyper et al., 2014) and our result.

### Interpretation using variance plot

**Fig. 2:**
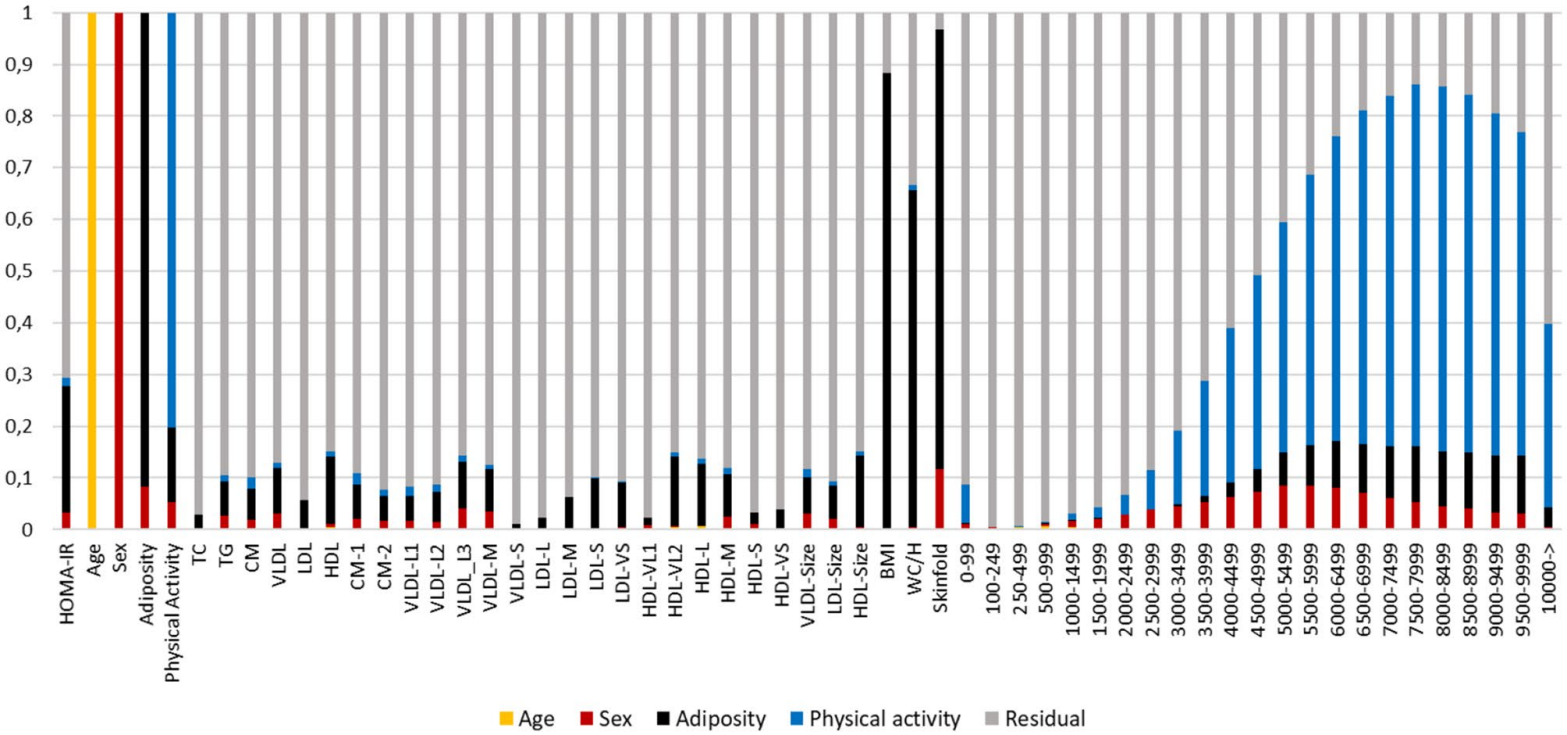
Variance plot showing the influence of covariates on outcome and explanatory variables

We have previously shown how variance plots can be used to visualize the influence of covariates on outcomes, explanatory variables, and each other (Rajalahti et al., 2021a). Figure 2 shows the variance for multiple covariate projections.

Covariate projections were done in the following order: Age, sex, adiposity, and PA. Color code: (i) age (yellow), (ii) sex (red), (iii) adiposity (black), and (iv) physical activity (blue). Residual variances after projections, which can be used for further modelling of net association patterns, are shown in grey. Projections for adiposity and PA used the target component scores which are also shown.

Due to narrow age range, age shares almost no variance with the other variables, while sex shares variance with the adiposity target component, skinfold, and PA with a maximum around 5500–6000 cpm. This is attributed to less PA among girls than boys in the analyzed cohort. The adiposity target component shares a variance pattern with the lipoproteins which was previously observed (Rajalahti et al., 2021a). The variance pattern shared between adiposity and PA complies with our findings above with increasing association with higher intensity PA. We also observe strong associations between adiposity and HOMA-IR whereas association of PA to HOMA-IR and lipoproteins are minor after adjustment by adiposity.

### Possible impact of residual covariate variance on models

**Fig. 3:**
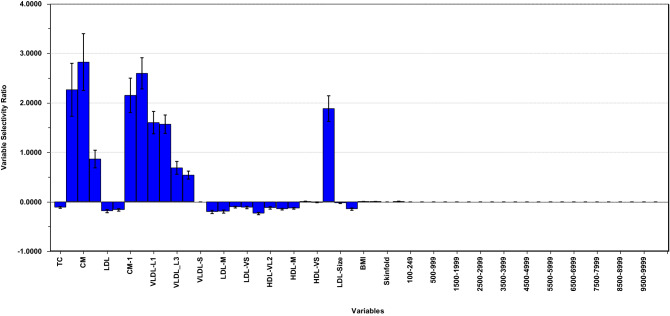
Selectivity ratio plot for HOMA-IR with adjusted variables included as explanatory variables

The variance plot shows that some adiposity measures and PA variables have considerable residual variance when using target components for covariate projections. To explore the possible impact of residual covariate variance in the regression model, we modelled HOMA-IR for the net data including the three adiposity measures and 23 PA variables together with the lipoproteins as explanatory variables. The SR plot (Fig. 3) displays neither significant associations to the adiposity measures BMI, WC/H, or skinfold nor to the PA variables. Furthermore, the association pattern of HOMA-IR to the lipoproteins is identical (Fig. 1d) to with explained variance in HOMA-IR being 9.0% compared to 8.9% (Table 1) for the corresponding model not including the adjusted adiposity and PA variables. This shows that our approach removes all the predictive information in the relation between outcome and covariates.

SR plot of regression model with HOMA-IR as outcome and the lipoprotein features, adiposity variables and the PA descriptor as explanatory variables. Data were adjusted for age, sex, adiposity, and PA prior to modelling.

## Conclusion

We developed a general approach to quantify and interpret the influence of strongly multicollinear and even linear dependent covariates on metabolomics association patterns explored by regression modelling. The method adjusts outcome and explanatory variables for covariates simultaneously and works irrespective of the number of covariates and their degree of mutual collinearity. Furthermore, our approach treats covariates as an integrated part of the model and thus acknowledges the complementary and important information supplied by these variables.

The present application using target projections to examine the influence of lifestyle related factors on the association pattern between HOMA-IR and a comprehensive lipoprotein profile, illustrates how the impact of covariates on association patterns can be quantified and interpreted. Their variance patterns provided additional insight into important aspects of the data and allowed for improved interpretation of etiology. Covariates should therefore be given a thorough examination in the modelling process.

Our modelling procedure incorporates validation and visualization tools to assure predictability and facilitate interpretation of association patterns.

## Supplementary Information

Below is the link to the electronic supplementary material.

Supplementary file1 (XLSX 449 kb)

Supplementary file2 (PPTX 48 kb)

## Data Availability

The data analyzed in this paper are available as supplementary material (Table S1).
