# Supplementary figures and images for: An approach to assess and adjust for the influence of multicollinear covariates on metabolomics association patterns—applied to a study of the associations between a comprehensive lipoprotein profile and the homeostatic model assessment of insulin resistance
Source: Metabolomics. 2022 Sep 2;18(9):72. doi: 10.1007/s11306-022-01931-6 (PMC9439979; doi:10.1007/s11306-022-01931-6)

## Slide 1
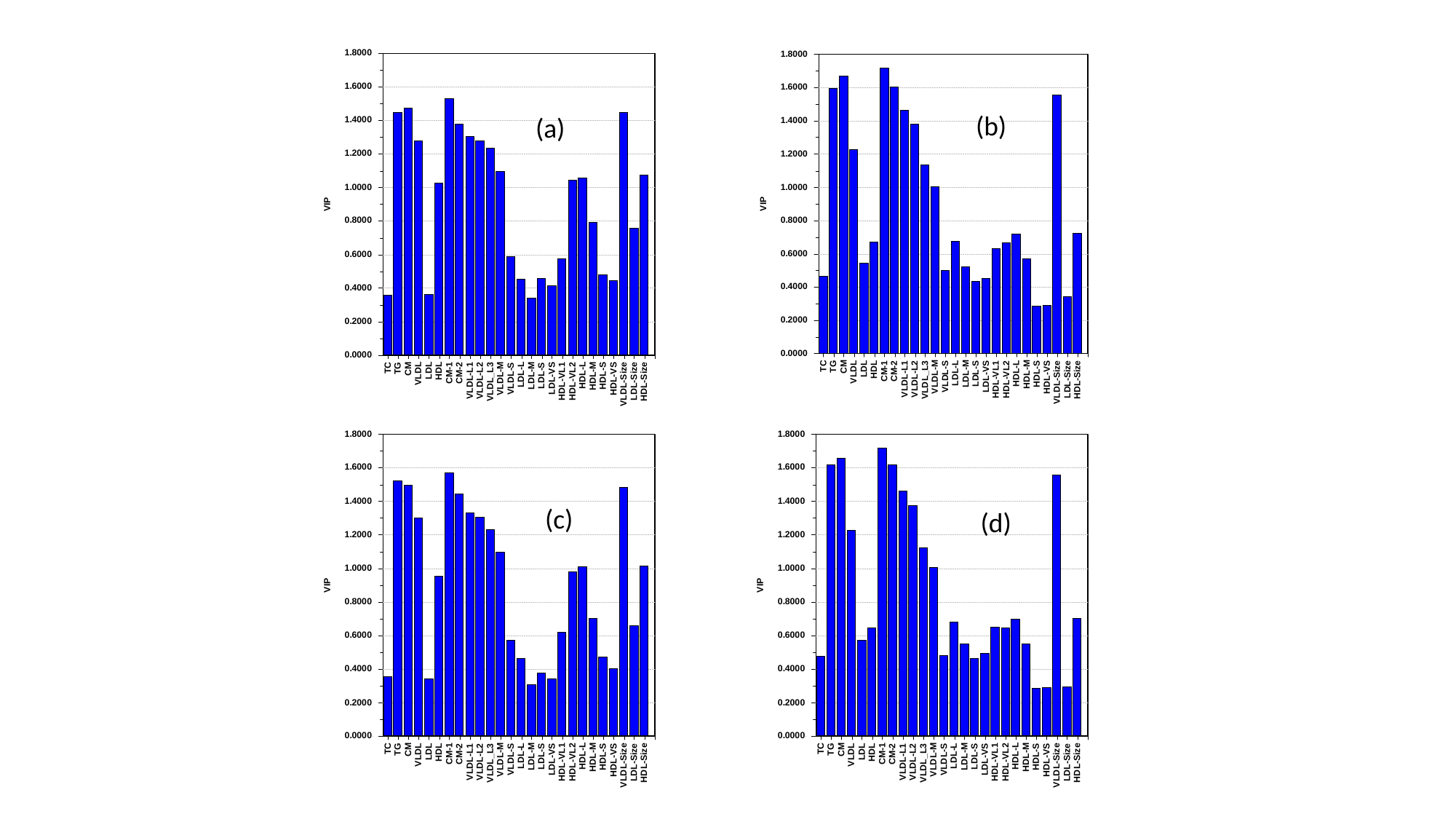

(b)
(a)
(c)
(d)

Supplement: Supplementary file 2 — Supplementary file2 (PPTX 48 kb) [file 11306_2022_1931_MOESM2_ESM.pptx]
